# South West Orthopaedic Club

**Published:** 1983-04

**Authors:** 


### South West Orthopaedic Club

Meeting with the Wessex Orthopaedic Club Saturday, 8th May 1982 at Weymouth

### THE DEVELOPMENT OF THE MÜLLER STRAIGHT STEM PROSTHESIS

P. Niederer, Protek, Switzerland

Their experience had shown that the weakest part of the prosthesis was the bone implant interface. Their studies had shown that there were defects in the laminations beneath the collar and beneath the lower part of the stem. They had developed the larger straight stemmed prosthesis. This provided better weight transmission and the large size of the prosthesis filled to a large part the cavity of the upper femur. This reduced the amount of cement filler which was necessary. This was illustrated on slides. They had tried uncemented prostheses but this as yet was not satisfactory. He demonstrated the various sizes and widths of stems available and the standard 32 mm to the small 22 mm for previous CDH patients. There is no collar on the femoral prosthesis but there is a small ridge which allows unity of the prosthesis and cement. Prosthetic replacement for the hip joint was designed not only to produce a new joint suface, but also if possible to restore leg length and alignment. It is therefore necessary sometimes to have a lateralised prosthesis up to 8 mm, this being necessary when there has been previous surgery on the femoral neck. The normal angle of the neck is 135° but for CDH patients the angle has been reduced to 130°.

The absolute necessity of pre-operative planning in terms of the size of the implant to fit the cavity and the length of the neck to restore leg length was stressed.

The reaming of the medullary canal was originally done with a powered reamer, but now a long stemmed rasp is used as this allows control of the rotation element and the slow movement of this rasp does not produce burning of the bone. He stated it was important when starting to ream the upper femur that the reamer must be posterior to the neck otherwise it may end up too anteriorly.

A plug of bone is taken from the removed femoral head to block the lower part of the femoral shaft and thus improve cement impaction.

Mr. Leigh then discussed 11 years experience of 426 patients in Exeter. Their femoral prosthesis differs from the Müller variety in that there is no collar and the lower part of the femoral stem is thinner. This allows a greater degree of elasticity and, he felt, places less stress on the bone. However, in the Exeter technique, particular stress is laid on the insertion of the cement. Their studies have shown that laminations did not occur around the prosthesis. Their study of patients some years after operation shows that actual subsidence clinically benign was also rare to a significant degree. He felt also that at least 4 or 5 mm of cement are necessary on the medial side of the prosthesis near the calcar. When inserting the cement, the surgeon's thumb covers the medial part of the upper femoral shaft and ensures penetration of the cement in this area.

H.R.

### LIGAMENTOUS INJURIES IN THE KNEE JOINT AND THEIR TREATMENT

E. L. Trickey, Royal National Orthopaedic Hospital

A rupture of the medial ligament alone would not require any surgical treatment unless the lower part is avulsed and there is opening of the joint with sucking in of the skin when a valgus stress is applied. In these cases the rupture is explored and the joint repaired. Wherever possible, the medial meniscus should be conserved. Ruptures of the medial ligament are often associated with tears of the anterior cruciate ligament and in these cases the Lachmans sign is positive. The jerk sign may not be positive if there is significant laxity on the medial side.

Mr Trickey felt that anterior cruciate ligament tears may be the predominant injury and these particularly occur in patients playing such sports as basketball. The patient often recalls a snap in the knee but is able to get up and try to play but is unable to continue to do so. There is always a hemipophysis in the knee. In these cases, Mr. Trickey advised arthroscopy to see where the cruciate ligament is ruptured. If the superior or inferior part is ruptured, then this may be repaired. If, however, the middle part is ruptured, then it is probably preferable to leave the injury and perform a secondary repair.

Ruptures on the lateral side of the joint are rarely solely a rupture of the lateral ligament and it is usually preferable to repair the damaged structures, except if the patient has a genual valgum when normally walking.

Ruptures of the posterior cruciate ligament are best repaired if there is an avulsion of a fragment of bone, but if within the substance of the posterior cruciate ligament, it is probably rarely necessary to repair these.

Chronic anterior medial instability is not a large problem, but rupture of the anterior cruciate ligament is important and Mr. Trickey felt that the pes anserinus transfer was no good. Where there is combined medial and anterior damage and anterior subluxation, he prefers the O'Donoghue procedure. For the anterior cruciate ligament, the McIntosh procedure abolishes the jerk but not the lateral instability. Mr. Trickey prefers the Kenneth Jones operation. He did note that this operation is often improperly performed and the patient is unable to fully flex the knee after operation. It is important that the reconstructed ligament goes posterior in the lateral condyle. Mr. Trickey illustrated how he did the operation, completely detaching the strip of patella tendon and rerouting it through a drill hole in the tibial condyle. The other end of the graft is then brought out through the intercondylar notch and over the top of the lateral femoral condyle, being sure that it passes far enough posterior in the knee joint. He always excises the interpatella fat pad as this makes the operation so much easier. He also noted that sometimes at arthroscopy, it may appear that the anterior cruciate ligament has remained attached. In these cases it is usually reattached to the posterior cruciate ligament. This repair abolishes the jerk and the Lachmans sign, the latter being the most important. Only if there is gross instability does he combine this with a McIntosh posterior lateral repair. He was unable to show any results as he felt that they were of little value unless they were at least five years after operation.

For posterior lateral subluxation, which is an external rotation of the tibia and may be seen on the lateral X-rays by the prominence of the head of the fibula, he does the Trillat operation. This is an advancement of the popliteus muscle and the lateral ligament and repair of the posterior lateral capsule.

H.R.

### SOME PITFALLS IN BONE SCANS

**S. Walkden, Weymouth**

Dr. Walkden, Consultant Radiologist at Weymouth Hospital, spoke on some pitfalls in bone scans. This was a very well illustrated talk showing a number of both negative and positive results in bone scan.

H.R.

### POSTMENOPAUSAL OSTEOPOROSIS

**M. J. Stevenson,**

Following the loss of ovarian function at the menopause, an acceleration of the natural bone loss of aging occurs in women. In a substantial proportion of women postmenopausal bone loss reduces the skeletal mass to below that expected for the age and sex — postmenopausal osteoporosis. The end result of osteoporosis is fracture after minimal trauma, the classical sites being spine, distal forearm and femoral neck. Thus postmenopausal women are affected four to five times more than men. More than one-third of women over the age of 65 suffer one of these fractures, and the cost to both patient and Health Service resources is horrendous.

The pathogenesis of postmenopausal bone loss has always been the subject of much debate. However, recent studies utilising direct measurement of bone-regulating hormones have shown that the increased bone resorption, which is responsible for the postmenopausal acceleration of bone loss, is mainly due to oestrogen deficiency which leads to a deficiency of calcitonin, a hormone whose function is that of skeletal protection. The minor changes that are found in vitamin D metabolites and calcium absorption are secondary to, rather than the cause of, the bone loss.

Prevention of bone loss is much more successful than treatment of established disease. It has now been shown beyond doubt that oestrogen replacement is the only therapy to prevent, and to some extent reverse, bone loss.

Whilst the diet should be adequate in calcium and vitamins, there is no convincing evidence that extra supplements of these are of benefit in the treatment of osteoporosis. Fluoride stimulates the formation of woven bone but is too toxic for widespread use, and suggested benefits are unproven in the long term. Recent studies suggest a beneficial effect of calcitonin treatment and confirmation is awaited.

With the correct use of hormone replacement therapy, unwanted uterine bleeding and serious side-effects can be eliminated. It is hoped that more effective combination treatments will be available in the near future. Orthopaedic surgeons are in a position to make early diagnosis of osteoporosis. The assistance of both endocrinologist and gynaecologist should be sought for the further management of the disease.

### THE BONE QUALITY DEFECT AND OSTEOPOROSIS IN PATIENTS WITH FRACTURE OF THE FEMORAL NECK: THEIR TREATMENT BY FLUORIDE, CALCIUM AND VITAMIN D

**I. G. Mackie and Z. A. Ralis, Cardiff**

A new histological defect in osteoporotic bone, in patients with femoral neck fractures, and their treatment with fluoride, calcium and vitamin D in a prospective controlled trial is described. The results indicate that this condition is now treatable.

### METHOD

Treatment started on 38 of 86 patients treated at the Cardiff Royal Infirmary with femoral neck fracture who were suggested for treatment because of their first 8 mm trephine iliac crest biopsy taken at the time of operation, indicated osteoporosis and/or the bone quality defect. They were given orally for 8 months Sodium Fluoride 60 mg on alternate days, Calcium Carbonate 1600 mg on alternate days and Vitamin D 1 mg per day as 1$\alpha$OH cholecalciferol. In a monthly clinic full physical examination, status of healing of the fracture and blood serum biochemistry were undertaken. After completion of treatment, under local anaesthetic the second iliac crest biopsy from the identical area in the contralateral iliac crest was taken from 23 patients (in six patients the therapy was discontinued as soon as they reported mild to moderate symptoms of gastric irritation and nine did not give consent to a second biopsy), together with the second set of radiographs of the thoracic and lumbar spine and of the second metacarpal for the cortical index measurements. These were compared with those taken before the treatment was commended. Histology in both biopsies was assessed in decalcified and undecalcified sections treated by the conventional staining techniques and in microradiographs, and in sections stained by the PTAIH and Tetrachrome methods. Bone mass was estimated by double blind counts separately for cortical and trabecular bone by the point counting technique. The same procedures were carried out in an untreated control group of 20 patients.

### RESULTS

There were no new vertebral or long bone fractures in the treatment group. There was also no significant increase in the mass of cortical bone as assessed from the metacarpal index on radiographs and in histological sections (the average increase was +18%). The trabecular bone mass, however, increased significantly in all 23 patients – it almost doubled the average increase to 92.8%. Simultaneously, there was also a significant improvement in the mineralisation of bone – 43% of patients almost doubled the area of the normally mineralised bone in 57% of them the increase was more than 100%, the average being 186%. These results not only confirmed the expected positive improvement of the trabecular bone mass after fluoride, calcium and Vitamin D treatment *but have also shown significant improvement in the quality of the previously defective bone and good mineralisation of the newly produced bone.*

The control group showed two new vertebral fractures and no change in the bone from the first biopsy.

### BRACING OR PLASTER FOR COLLES FRACTURES? A RANDOMISED PROSPECTIVE CONTROLLED TRIAL

**G. C. Bannister and A. G. F. Gibson, Bristol**

Appraisal of the literature suggests that the functional result of Colles' fracture is dependent upon the anatomical position of the fracture at union. The vast majority of Colles fractures are managed by closed methods but it has been long known that up to 60% of cases so treated slip back to their original position. Some 30% of cases have permanent problems of function after Colles' fracture and the lowest incidence of these (7%) has been reported by Sarmiento who treated his cases by functional bracing in supination. In this series only 12% lost anatomical position.

To evaluate whether improved function from the supinated brace was the result of good anatomical position or early movement 108 displaced Colles' fractures were treated by closed reduction under regional anaesthesia and immobilisation in a supinated brace (Group I), a supinated long arm cast (Group II) and a pronated short arm cast (Group III). If supination were to give good anatomical results Groups I and II would be expected to have better function than Group III. If early movement were the critical factor better function would be anticipated in the braced group.

The criteria for anatomical assessment were those of Lidström and for function of Gartland and Werley.

From the functional aspect at nine weeks after the fracture 90% of patients braced had satisfactory results compared with 50% of those treated in plaster. On discharge at 20 weeks there were 95% satisfactory results from bracing and 84% from treatment in plaster. Pronation was more easily regained in those immobilised in supination (94%) than supination in patients treated in pronated casts (54%).

From the anatomical standpoint position was satisfactorily maintained in 52% of patients immobilised in supination and 38% held in pronation. By all methods satisfactory anatomical position afforded 80% good function whilst unsatisfactory position realised 61% satisfactory function. Anatomical position was held more satisfactorily in the younger age group (60%) then in the over 50 age group (42%).

It would appear that early movement gives better functional results in the closed treatment of Colles' fracture.

### AUSTIN MOORE PROSTHESIS IN TREATMENT OF FRACTURE NECK OF FEMUR

**Maher Halawa and H. Abdelazeem, Plymouth**

One hundred and thirty-three fractures of neck of femur in 129 patients underwent hemiarthroplasty using Austin Moore prosthesis in Mount Gould Hospital in the period from June 1978 to January 1980. This Paper deals with the evaluation of the notes of 81 patients, and follow-up of 48 patients clinically reviewed for an average period of 21 months.

Of the patients 42.1% could not walk after surgery. The majority of them were due to operative and postoperative complications. Our dislocation rate in this series was 12.78%. It was quite alarming that 75.8% of these ended fatally, and 58.8% needed removal of the prosthesis.

We do not recommend the use of Austin Moore prosthesis in the treatment of fractured neck of femur in the elderly. A cemented Thompson is more advisable, and the posterior approach should be abandoned for this type of patient. A modified McFarland Osborne approach is recommended.

### SWANSON PROSTHESIS VERSUS KELLERS OPERATION FOR HALLUX VALGUS OR HALLUX RIGIDUS

**J. D. Wrighton and N. J. Stalley, Weymouth**

A prospective clinical trial was performed on patients with hallux valgus and hallux rigidus taking alternate toes with Keller's or Swanson's prosthesis.

Fifty four operations were performed by the same surgeon and follow up review was performed by the Junior Staff. There were four groups of toes, hallux valgus or rigidus with Keller's or Swanson Silastic prostheses.

The number of patients treated was 36, 31 females, 5 males, aged between 27 and 77.

The total number of feet treated was 44, 30 hallux valgus and 14 hallux rigidus. Duration of pain was for at least one year and the site of pain was the metatarsophalangeal joint.

### RESULTS

*Bunion formation*. In the hallux valgus group 28 had a bunion and at review one was present in the Keller's and one with the prosthesis. In the hallux rigidus group, 7 had bunions pre-operatively, nil at review.

*Metatarsalgia*. This was present in all groups before surgery and there was an increase in metatarsalgia after surgery which was less marked with the prosthesis used for hallux rigidus.

*Walking distance*. Patients were asked if they could walk a mile before and after surgery and there was very little difference between the prosthetic and Keller's results.

*Postoperative recovery*. Patients were asked how long it took to wear normal shoes and at three months this was equivocal in each group.

*Satisfaction*. The results in this small series were good (90%), the prosthesis being more popular.

*Reasons for dissatisfaction*. Five patients were dissatisfied, 4 Kellers and one prosthetic. Their reasons were: (1) Post-op deep infection requiring the prosthesis to be removed. (2) In two cases with the Kellers in comparison with the prosthesis in the other foot, the toe being shorter and more floppy. The two other cases had pain in the joint.

*Valgus deformity*. The valgus deformity was better corrected by Kellers operation than by using a prosthesis, but the mobility was better using a prosthesis.

*Bilateral cases*. There were six patients with bilateral hallux valgus and one hallux rigidus. Of these cases four found no difference between surgery, two preferred the prosthesis and one the Keller's.

### CONCLUSIONS

1. Both operations are effective at relieving the symptoms of pain at the metatarsophalangeal joint.
2. The prosthesis is less effective at correcting the valgus deformity.
3. The metatarsophalangeal joint is more mobile using the prosthesis than with Keller's.
4. Infection is a risk using a prosthesis.
5. More patients were satisfied with the prosthesis than the Keller's, owing to the fact that there was less loss of length to the hallux and less pain.

### WHEN TO USE THE PROSTHESIS

The best indications are in hallux rigidus and mild to moderate hallux valgus.

### THE LONG TERM RESULTS OF PELVIC OSTEOTOMY IN ADULTS WITH HIP DYSPLASIA

**D. A. P. Ainscow, Southampton**

The long term results of pelvic osteotomy for hip dysplasia in 38 adults (50 hips) are presented after an average interval of 10 years. The average age of the patients was 36 and they were divided into two groups; Group A were those with no evidence of degenerative change before operation and Group B, those who had degenerative change before operation.

The operation performed consisted of a pelvic osteotomy performed through a routine sup periosteal approach with preliminary division of the psoas tendon through a separate incision at the lesser trochanter. The acetabulum was redirected anteriorally and laterally and the position was maintained with a bone graft secured by stainless steel pins.

The hips were assessed radiologically before and after operation, and clinically using a combination of the methods suggested by Thompson and Charnley. Based on the clinical assessment, the hips were allocated to one of four groups; excellent, good, fair and poor. In Group A there were 23 hips, seven of which were fair and 16 poor before operation. The chief complaints of the patients were painful locking and giving way of the joint and a Trendellenburg gait. Eight patients required walking sticks. After operation there were five excellent, 12 good and four fair hips. Only one patient required a walking stick after operation and there was no evidence of deterioration of the clinical results with the passage of time. Two hips in this group were unsatisfactory and required further surgery.

In Group B there were 27 hips, of which nine were fair and 18 poor before operation. At the end of review there were five good, eight fair and one poor result. Thirteen patients had undergone total hip replacement without complication. The average period of pain relief was 7.8 years (range 4 to 11 years) and the average time for conversion to total hip replacement was 8.6 years after pelvic osteotomy.

The radiographic assessment showed that subluxation of the hip was reduced in all patients, Shenton's line was restored in 19 hips and the discontinuity reduced in 11 hips. The centre edge angle of Wieberg was improved from an average of 10 degrees before operation to 25 degrees after operation, and the adult acetabular index was reduced from an average of 51 degrees before operation to 42 degrees after operation.

In the hips without degenerative change before operation there was no evidence of degenerative change developing during the period of follow-up. In the hips in Group B, where degenerative change was present, there was no evidence of reversal of these changes but the rate of progression slowed.

It is suggested that this form of pelvic osteotomy can lead to a permanent improvement in suitable hips if performed before degenerative change has occurred. Once degenerative change has occurred an average period of pain relief of 7.8 years was achieved by this operation. By improving the antrolateral cover of the acetabulum subsequent total hip replacement is facilitated.

